# A Dried Blood Spot protocol for high-throughput quantitative analysis of SARS-CoV-2 RBD serology based on the Roche Elecsys system

**DOI:** 10.1128/spectrum.02885-23

**Published:** 2024-03-01

**Authors:** Noemi Castelletti, Ivana Paunovic, Raquel Rubio-Acero, Jessica Beyerl, Michael Plank, Christina Reinkemeyer, Inge Kroidl, Ivan Noreña, Simon Winter, Laura Olbrich, Christian Janke, Michael Hoelscher, Andreas Wieser, on behalf of the KoCo19/ORCHESTRA Working group

**Affiliations:** 1 Division of Infectious Diseases and Tropical Medicine, LMU University Hospital, LMU Munich, Munich, Germany; 2 Institute of Radiation Medicine, Helmholtz Zentrum München, Neuherberg, Germany; 3 Fraunhofer Institute for Translational Medicine and Pharmacology ITMP, Immunology, Infection and Pandemic Research, Munich, Germany; 4 Max-von-Pettenkofer Institute, LMU Munich, Munich, Germany; 5 German Center for Infection Research (DZIF), partner site Munich, Munich, Germany; 6 Center for International Health (CIH), University Hospital, LMU Munich, Munich, Germany; Universidade Federal do Rio de Janeiro, Rio de Janeiro, Brazil

**Keywords:** COVID-19, SARS-CoV-2, DBS, antibody, serology, Roche Elecsys, Spike, quantitative

## Abstract

SARS-CoV-2 spreads pandemically since 2020; in 2021, effective vaccinations became available and vaccination campaigns commenced. Still, it is hard to track the spread of the infection or to assess vaccination success in the broader population. Measuring specific anti-SARS-CoV-2 antibodies is the most effective tool to track the spread of the infection or successful vaccinations. The need for venous-blood sampling however poses a significant barrier for large studies. Dried-blood-spots on filter-cards (DBS) have been used for SARS-CoV-2 serology in our laboratory, but so far not to follow quantitative SARS-CoV-2 anti-spike reactivity in a longitudinal cohort. We developed a semi-automated protocol or quantitative SARS-CoV-2 anti-spike serology from self-sampled DBS, validating it in a cohort of matched DBS and venous-blood samples (*n* = 825). We investigated chromatographic effects, reproducibility, and carry-over effects and calculated a positivity threshold as well as a conversion formula to determine the quantitative binding units in the DBS with confidence intervals. Sensitivity and specificity reached 96.63% and 97.81%, respectively, compared to the same test performed in paired venous samples. Between a signal of 0.018 and 250 U/mL, we calculated a correction formula. Measuring longitudinal samples during vaccinations, we demonstrated relative changes in titers over time in several individuals and in a longitudinal cohort over four follow-ups. DBS sampling has proven itself for anti-nucleocapsid serosurveys in our laboratory. Similarly, anti-spike high-throughput DBS serology is feasible as a complementary assay. Quantitative measurements are accurate enough to follow titer dynamics in populations also after vaccination campaigns. This work was supported by the Bavarian State Ministry of Science and the Arts; LMU University Hospital, LMU Munich; Helmholtz Center Munich; University of Bonn; University of Bielefeld; German Ministry for Education and Research (proj. nr.: 01KI20271 and others) and the Medical Biodefense Research Program of the Bundeswehr Medical Service. Roche Diagnostics provided kits and machines for analyses at discounted rates. The project is funded also by the European-wide Consortium ORCHESTRA. The ORCHESTRA project has received funding from the European Union’s Horizon 2020 research and innovation program under grant agreement No 101016167. The views expressed in this publication are the sole responsibility of the author, and the Commission is not responsible for any use that may be made of the information it contains.

**IMPORTANCE:**

SARS-CoV-2 has been spreading globally as a pandemic since 2020. To determine the prevalence of SARS-CoV-2 antibodies among populations, the most effective public health tool is measuring specific anti-SARS-CoV-2 antibodies induced by infection or vaccination. However, conducting large-scale studies that involve venous-blood sampling is challenging due to the associated feasibility and cost issues. A more cost-efficient and less invasive method for SARS-CoV-2 serological testing is using Dried-Blood-Spots on filter cards (DBS). In this paper, we have developed a semi-automated protocol for quantifying SARS-CoV-2 anti-spike antibodies from self-collected DBS. Our laboratory has previously successfully used DBS sampling for anti-nucleocapsid antibody surveys. Likewise, conducting high-throughput DBS serology for anti-spike antibodies is feasible as an additional test that can be performed using the same sample preparation as the anti-nucleocapsid analysis. The quantitative measurements obtained are accurate enough to track the dynamics of antibody levels in populations, even after vaccination campaigns.

## INTRODUCTION

The severe acute respiratory syndrome coronavirus 2 (SARS-CoV-2) causing COVID-19 was reported for the first time on 31 December 2019 in the city of Wuhan (Hubei province, China) in pneumonia patients (1). Subsequently, pandemic spread was observed leading to more than 767 million infected individuals and in excess of 6.9 million fatalities as of 7 June 2023 (2). The pandemic has exposed dramatic capacity limitations in the healthcare and laboratory/diagnostic sectors in many countries (3). Despite huge efforts and many different quickly developed test systems, tracking the spread of the disease in the population remains a huge challenge (4). In 2021, vaccinations became increasingly available in many countries, leading to reduced death rates due to COVID-19 (5). With increasing vaccination rates as well as ongoing transmission of SARS-CoV-2 and its variants, understanding the spread of the disease as well as the duration and effectiveness of the vaccines in the population is crucial (6). High-throughput serological assays are capable of providing valuable information on the quantitative serostatus for many subjects in a short time (7). However, serological parameters are generally measured in venous blood, and the collection of blood from a large number of individuals is challenging due to the relatively high cost and the medical personnel needed to obtain the samples (8). Even such constraints are more pronounced in resource-limited settings (9). Countries with elaborate healthcare systems also struggle to establish good sero-epidemiological data of their population over time (10). Hence, the establishment of high-throughput serology tests relying on self-sampling would be a highly effective tool. Thereby, it would be desirable to obtain reliable and quantifiable serologic parameters to estimate the level of protection. Furthermore, it is of special interest to obtain quantitative anti-spike (anti-S) responses as well as at least qualitative but sensitive anti-nucleocapsid (anti-N) titers to differentiate between vaccination-induced antibody responses and titers secondary to natural infection or a mixture of vaccination and potentially recent infections (11).

Dried-blood-spot (DBS) specimen collection offers a convenient method for large-scale population screening programs due to its less invasive nature compared to venipuncture. It also eliminates the need for medical personnel to collect samples. Once dried, DBS samples remain stable for extended periods under diverse conditions (12) further enhancing their suitability for long-term storage and transportation.

DBS sampling has already been used in large-scale population screening programs (13–15). In a study by Roxhed et al., a multianalyte and complex approach was applied to 878 randomly selected, undiagnosed home-sampled DBS collected in spring 2020 in Sweden (15). The cutoff point was established at more than six times the SD in relation to the most prevalent antibody level identified among the probable negative controls. In another investigation by Nikiforuk et al., a multiplex anti-immunoglobulin (IgG) assay was employed to analyze 6,841 samples collected from non-hospitalized children and adults in British Columbia, Canada, between 2020 and 2021 (16). The study demonstrated that in the unvaccinated population, the sensitivity and specificity of DBS testing were 79% (95% CI: 58%–91%) and 97% (95% CI: 95%–98%), respectively. However, in the double-vaccinated population, the sensitivity increased to 100% (95% CI: 88%–100%). Demonbreun et al. focused on comparing nucleic acid amplification (RT-PCR) tests and serological tests for detecting past SARS-CoV-2 infections (17). They analyzed 7,935 samples collected between June and December 2020 from the metropolitan area of Chicago in the United States. The study revealed that serology identified seven times more prior infections compared to confirmed SARS-CoV-2 RT-PCR.

With the beginning of the ongoing pandemic and the need to follow up the serology of our own cohort (18–22), we established a DBS-based highly reliable anti-N serology (18). In this manuscript, we present a quantitative anti-S serology based on the Elecsys anti-SARS-CoV-2 S assay (09289275190, Roche) that we use within the cohort since spring 2021. We demonstrate not only high sensitivity (96.63%) and specificity (97.81%) as compared to the same assay performed from venous blood samples on the same day with the same batch but also the feasibility to perform assessments of titer changes over time in individuals after vaccination and on a complete cohort over four follow-ups. The results are calculated as binding antibody units in venous blood (BAU) to deliver comparable and reliable titer information. Our protocol is also harmonized with the aforementioned anti-N serology and can be performed from the same sample eluate in one analysis run on an e801 analyzer, allowing for high throughput as well as cost efficiency.

## MATERIALS AND METHODS

### Patient samples

Participant’s samples for establishing and validating the method were obtained within a randomly selected population-based COVID-19 cohort study (KoCo19) in Munich, Germany which is part of the European-wide Consortium ORCHESTRA. The primary results and the selection process are described elsewhere (19–21). In brief, in a longitudinal population-based cohort and their sub-cohorts, subjects were recruited between the first sampling round in March/April 2020 to winter 2022. DBS and venous blood samples were obtained on the same occasion as matching pairs for evaluation of the performance of the DBS samples. Matching plasma samples were collected in EDTA-coated tubes and maintained at 4°C until centrifugation. Plasma samples were stored at −80°C in temperature-controlled biobank freezers. DBS samples were obtained from capillary blood with the help of single-use safety lancets (Sarstedt safety 85.1016). The blood was dropped onto barcode labeled neonatal screening filter cards (8.460.0004 .A Rev.1, Ahlstrom-Munksjö) and left to air-dry for 12–24 hours at room temperature protected from direct sunlight. Filter paper cards were stored dry at −80°C or 4°C in plastic boxes until they were used for analysis. Finally, 100 sample pairs (50% positive) were used for the establishment of the protocol, which was then validated using 725 additional matched samples. Other groups of samples were obtained only by DBS home sampling as described previously (6). As home sampling involves the drawing of capillary blood at home with a CE marked kit and a written as well as video instruction (https://www.youtube.com/watch?v=vpZUzuQV10E&feature=emb_title), some subjects were advised not to perform the sampling. Pregnant women as well as those subjects with anticoagulant therapy or known bleeding disorders were advised not to perform sampling due to slightly higher risk, such as prolonged bleeding or fainting. The KoCo19 cohort was subsequently followed up on five occasions. In this analysis, we present the evolution of their anti-N and anti-S antibodies from the second follow-up (March 2021, during the peak of the third wave in Munich and the early stages of the vaccination campaign for the general population) to the fifth follow-up (November 2022, marking the onset of the Omicron wave).

Additionally, anonymized DBS cards from colleagues and other study participants were included in this study. The participants received blood collection sets (Euroimmun ZV 9701–0101) by mail and were asked to prepare the DBS at home and send back the inoculated screening cards. Instructions for use, as well as a link to a video tutorial (https://youtu.be/vpZUzuQV10E) were provided. Healthcare professionals included in the study as colleagues or participants sometimes performed DBS collection at work. Upon reception of the DBS cards in the laboratory, barcodes were scanned, and samples were stored at 4°C until analysis within the next 48–72 hours.

### DBS quality control

In the blood collection for the DBS, we requested that at least two of the five circles on the filter paper card were completely soaked with blood. Detailed instructions were provided to dry the DBS at room temperature for 12–24 hours protected from direct sunlight before packing and shipping to the laboratory. In this study, we have also investigated the most common concerns related to the inhomogeneity of blood contents across DBS samples, like the coffee stain and the volcano effects, which describe a higher concentration at the edge of a DBS or in the center, respectively (23). Besides, we investigated the reproducibility and carry over effects between samples and uncertainties associated with back-calculation to BAU/mL as measured in venous samples.

### Extraction of antibodies from the DBS

To achieve a high sample throughput that allows us to measure anti-N and anti-S serology, we optimized the semi-automated workflow reported elsewhere (18). In brief, three discs with 3.2 mm in diameter per DBS sample were punched with a Panthera-Puncher 9 Instrument (PerkinElmer). The position of the discs is automatically assigned by the machine if not indicated otherwise. Discs are collected in a barcoded 96-well plate. 80 µL of elution buffer per well was dispensed with a JANUS G3 workstation (PerkinElmer) with eight channels, and elution was performed in a temperature-controlled shaker (MIUlab ES-60E) for 1 hour at 37°C and 300 rpm. After elution, microtiter plates were transferred into a workstation where 62.5 µL of each eluted blood/buffer solution is transferred to a tray with 15 individually barcoded 5-rack packs of the Roche e801 machine. Roche 13/16 micro sample cups (Roche, 05085713001) were used to minimize the dead-volume needed for analysis in the Roche Elecsys system. Alternatively, sample transfer was performed with adjustable tip spacing multichannel pipettes (Integra Voyager) to perform the process manually.

### Roche Elecsys anti-SARS-CoV-2 S immunoassay

The Roche Elecsys anti-SARS-CoV-2 S assay uses a recombinant protein representing a truncated Spike (S) antigen in a double-antigen sandwich assay format, detecting antibodies of all subclasses against SARS-CoV-2. The immunoassay was performed on a Cobas e801 analytical unit (Roche) using the electrochemiluminescence (ELECSYS) technology. Roche Elecsys anti-SARS-CoV-2 S is a quantitative assay validated for use with human serum and plasma. The results are given in Units/mL, and for venous blood are equivalent to the standardized BAU. In this study, extensive validation was performed to provide quantitative readouts of the S1/RBD responses using the raw values obtained by the assay. DBS and venous blood matching pairs were measured on the same day with the same batch of ELECSYS Kit.

### Statistics

Prior to analysis, we cleaned and locked the data. For the analyses and visualization, we used the software R, version 4.0.5. In operational replicates for DBS, we used the lowest value in the linear range of the assay with the lowest dilution. Dilutions were performed if the DBS eluate or the plasma sample yielded values above 250 U/mL as this is outside the linear range of the assay. Dilution factors were back-calculated to reveal the true concentration of the samples. In case plasma samples of the same dilution were measured multiple times, the last measurement was chosen. The cutoff value was empirically defined with an establishment data set of 100 matched DBS/plasma samples (50% positives). The number of false positives/negatives was minimized. Sensitivity and specificity were calculated for each set of cutoffs referring to the plasma result, giving the percentage of samples positive or negative, respectively, in both DBS and plasma. For association among continuous variables, we report the Spearman’s correlation. For multiple group comparisons, we applied Kruskal-Wallis tests. The correction formula $venousUnits=10^{intercept+slope\cdot log_{10}DBSUnits}$ to calculate back plasma values from raw DBS measurements was derived using the ordinary nonparametric bootstrap method using 10,000 nonparametric bootstrap replicates to calculate the mean and the adjusted bootstrap confidence (BCA) interval (95% level) for slope and intercept (24–26).

### Data handling

DBS cards were individually barcoded within the study; barcodes were automatically scanned by the puncher. The puncher automatically links the barcode with a position on the 96-well plate into which the punched paper discs are automatically dispensed. Each 96-well plate is uniquely identifiable with another barcode attached to the plate. The software is adjusted to reject punching the same barcoded filter paper twice in the same plate. Data are transferred to the Janus robot using a self-programed script. Eluates are loaded into micro sample cup inserts in barcoded rack packs in groups of five. In parallel, a worklist for the Cobas analyzer is generated which is transferred to allow automated analysis as well as result export including luminescence signal values matched to the respective barcode of the DBS sample.

## RESULTS

**Fig 1:**
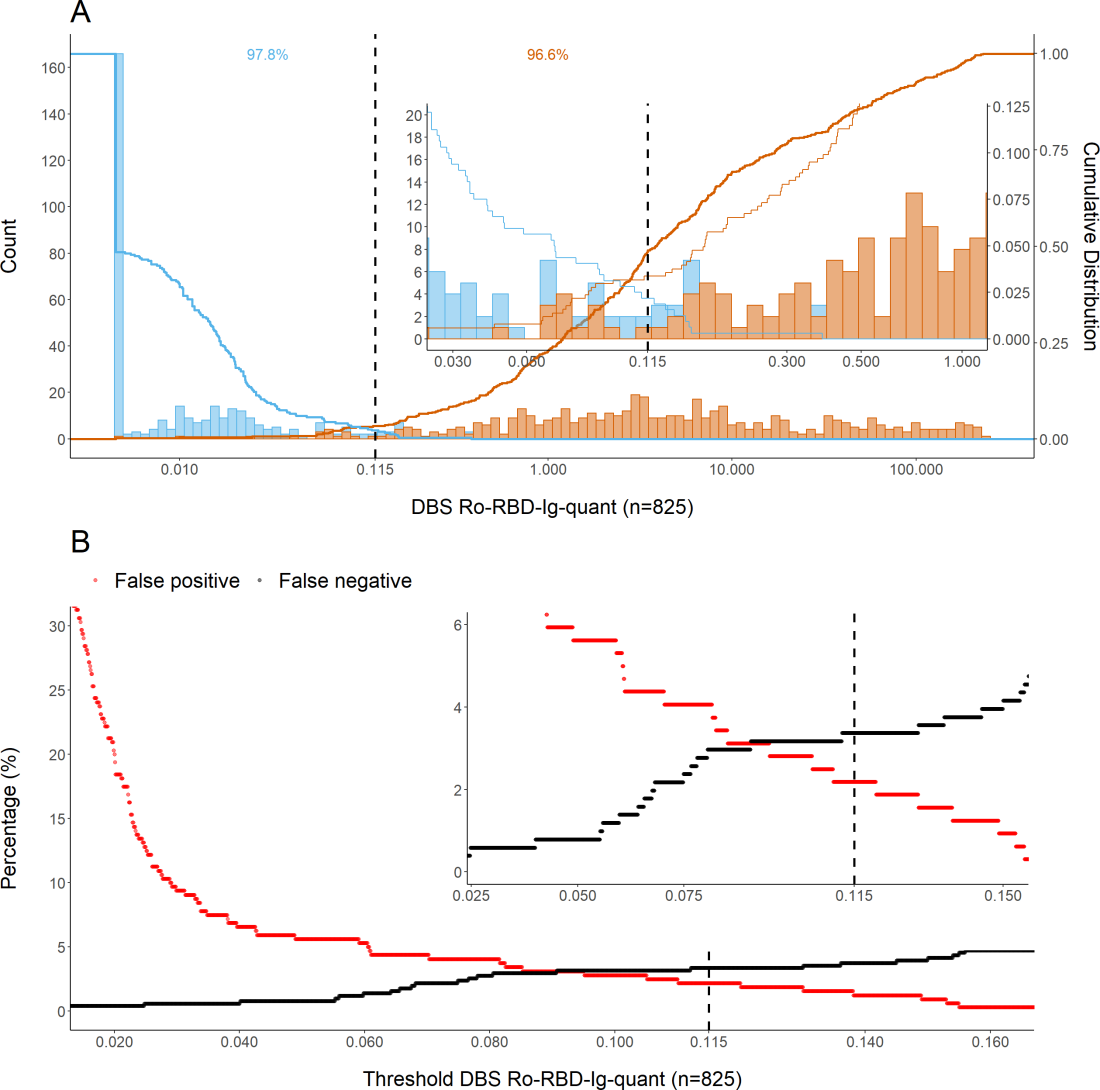
Description of the data. DBS eluates from patients of our in-house KoCo19 cohort and volunteer donors (*n* = 825) are used. The dashed vertical line denotes the empirically determined cutoff value (0.115) for result classification. (**A**) Frequency distribution of detected antibody levels against SARS-CoV-2 spike/RBD in vaccinated or naturally infected individuals. The insert in the top right represents a zoom-in on the y-axis to allow visualization of the cutoff region. (**B**) Empirical cutoff determination (dashed vertical line). Percentages of false positives/negatives depending on a variable threshold for DBS are shown in red or black, respectively. The cutoff was chosen to minimize false positive results.

DBS on filter paper has been used for SARS-CoV-2 serology studies successfully in our laboratory (18). In this study, DBS/venous blood sample pairs from the same patient at the same time point were measured using the Elecsys anti-SARS-CoV-2 S assay (measured on the same day with same batch) and used to establish cutoff values as well as the formula for calculating the quantitative venous values based on DBS results. We started with a set of 100 pairs including 50 positive samples to establish a cutoff value (Fig. S1). This was confirmed with further 725 paired samples. Positive and negative populations were found to be well-separated populations when analyzing the whole data set (Fig. 1A), and the cutoff was determined empirically at the value of 0.115 using the establishment data set. Thereby, optimizing sensitivity and specificity, with a special focus on avoiding false positive results (Fig. 1B).

**Fig 2:**
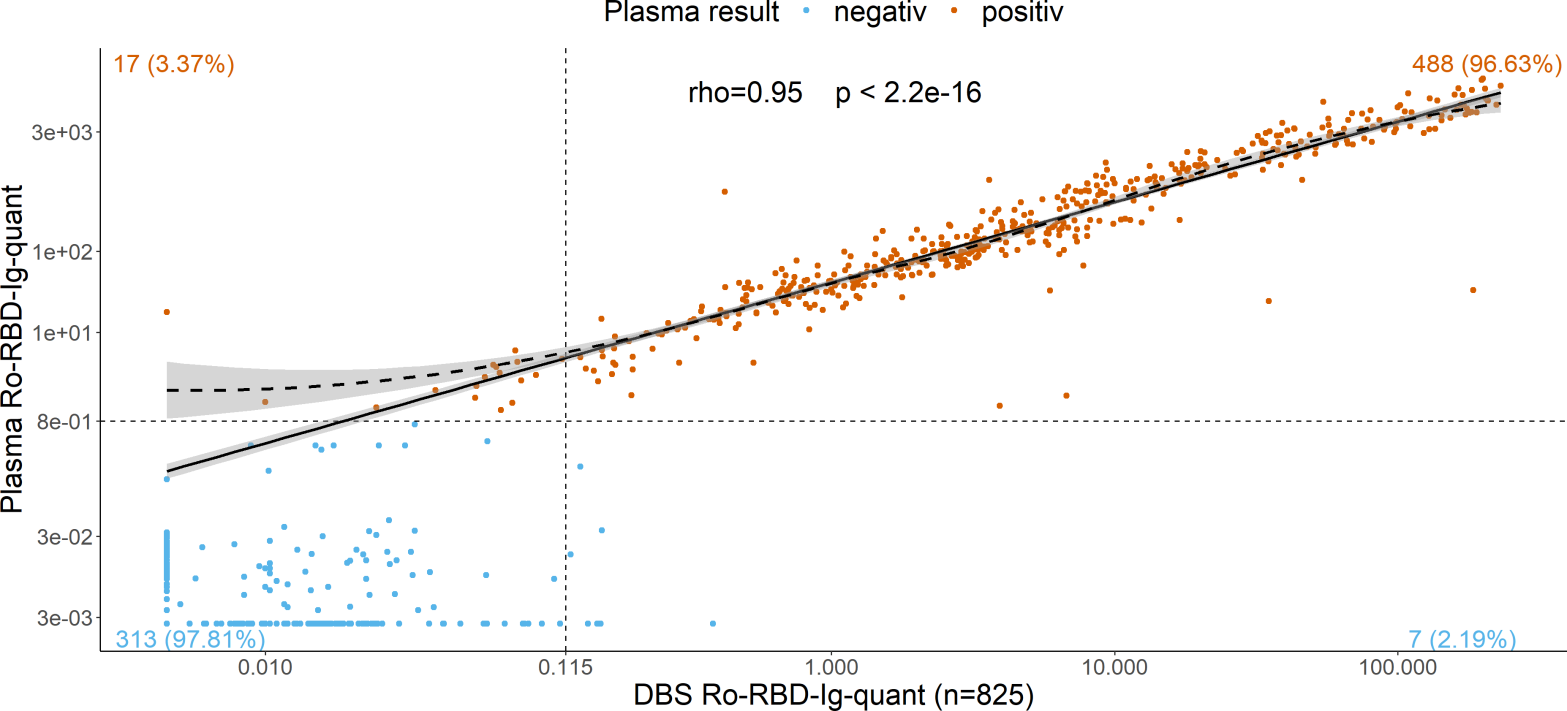
Scatterplot illustrating the linear relation on log10–log10 scale between antibody units detected in the DBS eluate (x-axis) and the corresponding venous plasma (y-axis) (*n* = 825). Correlation is calculated using the Spearman method. The dashed horizontal line denotes the manufacturer’s cutoff value for plasma result classification (0.8), while the dashed vertical line is the empirically determined cutoff value for DBS result classification (0.115). The solid black line represents a linear regression, while the dashed line is the LOESS (locally estimated scatterplot smoothing or local regression) modeling different types of associations. The gray region is the 95% CI of the linear/LOESS estimate.

This choice was made as the primary objective of the assay will be the assessment of vaccination titers which generally are very high and not close to any cutoff and will thus not be missed. The establishment data set is derived predominantly from naturally infected individuals. On the other side, a false negative result with a very low titer would be indicative of insufficient reaction to a vaccine, and this will be true for negative or very low titers alike. So we decided to accept a higher error rate in this direction due to the consequences. As the reference for the measurement of the DBS eluate, the plasma value of the same patient on the same day was used (Fig. S2). From the 825 paired samples measured, we found that 2.19% (7/320) were false positive, and 3.37% (17/505) were false negative, resulting in a sensitivity and specificity of 96.63% and 97.81%, respectively (Fig. 1A and 2).

To investigate the possibility for the quantitative measurement of antibody titers using DBS eluates, we determined all values of the paired samples in a quantitative manner. Thus, all eluates or venous plasma samples were diluted with Diluent Universal 2 (Roche, Mannheim, Germany), in steps of 1:10, 1:100, and 1:1000, where appropriate, to yield values within the linear measuring range of the assay which is specified below 250 Units/mL. Original concentrations were subsequently back-calculated based on the diluted value and the dilution factor. Quantitative results of the DBS eluates and the plasma values were correlated on a log10/log10 scale (Fig. 2). The correlation was found to be high (Spearman’s rho = 0·95). Applying the ordinary nonparametric bootstrap method, the correction formula $venousUnits=10^{1\cdot 623+0\cdot 986\cdot log_{10}DBSUnits}$ to calculate back plasma values from raw DBS measurements could be derived [adjusted bootstrap percentile (BCA) intervals, calculated with 10,000 replicates, of intercept and slope are (1.590; 1.695) and (0·900; 1·022), respectively]. See Fig. S3 for model evaluation.

**Fig 3:**
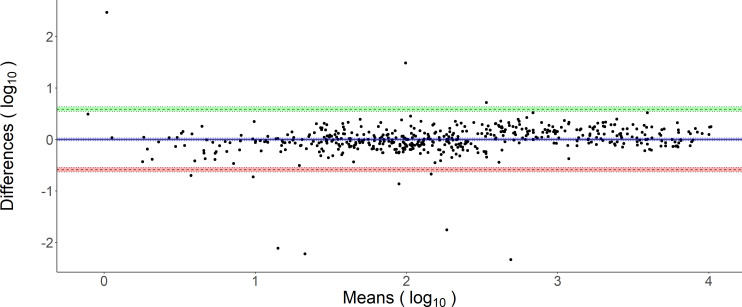
Bland-Altman plot illustrating the differences as a function of means for discrepancies between plasma-values and DBS-values converted to plasma units. Both plasma and converted DBS values are displayed as log10 scales for the analysis. The mean value signal difference is ${3\cdot 39\cdot 10}^{-15}$ BAU with 95% of the values in the interval (−0.588 BAU; +0.588 BAU).

To assess the variation and the accuracy of the quantitative DBS measurements compared to the plasma values as the gold standard, we visualized the data points in a Bland-Altman diagram (Fig. 3). The mean difference was found to be very small, 1 BAU with 95% of the values in the interval (0.258 BAU; 3.872 BAU).

To allow home sampling and uncomplicated shipping of DBS cards by mail, the preanalytical stability of the titer values has to be guaranteed. DBS cards were generated and subjected to different conditions that might be encountered due to the handling by ordinary subjects in preanalytics. Care was given to produce clear instruction materials as well as a training video. Still, the conditions (i) wet packaging (less than 3 hours of drying time) and (ii) sunlight (a whole day in direct sunlight on the window board at room temperature) were included in the evaluation. Additionally, the temperature conditions room temperature (22°C–25°C), fridge (4°C–7°C), freezer (−18°C–21°C), and incubator (36°C–38°C) were evaluated for 11 consecutive days after the sampling day to cover possible delays in shipping or posting the sample. No significant deterioration was observed in the groups stored in the freezer, in the fridge, and at room temperature (see Fig. S5). For 37°C and direct sunlight as well as wet papers for 11 days, a slight significant decrease was observed. Shorter exposure to such conditions leads to a significant decay only for sunlight after already 9 days, for both anti-N and anti-S.

**Fig 4:**
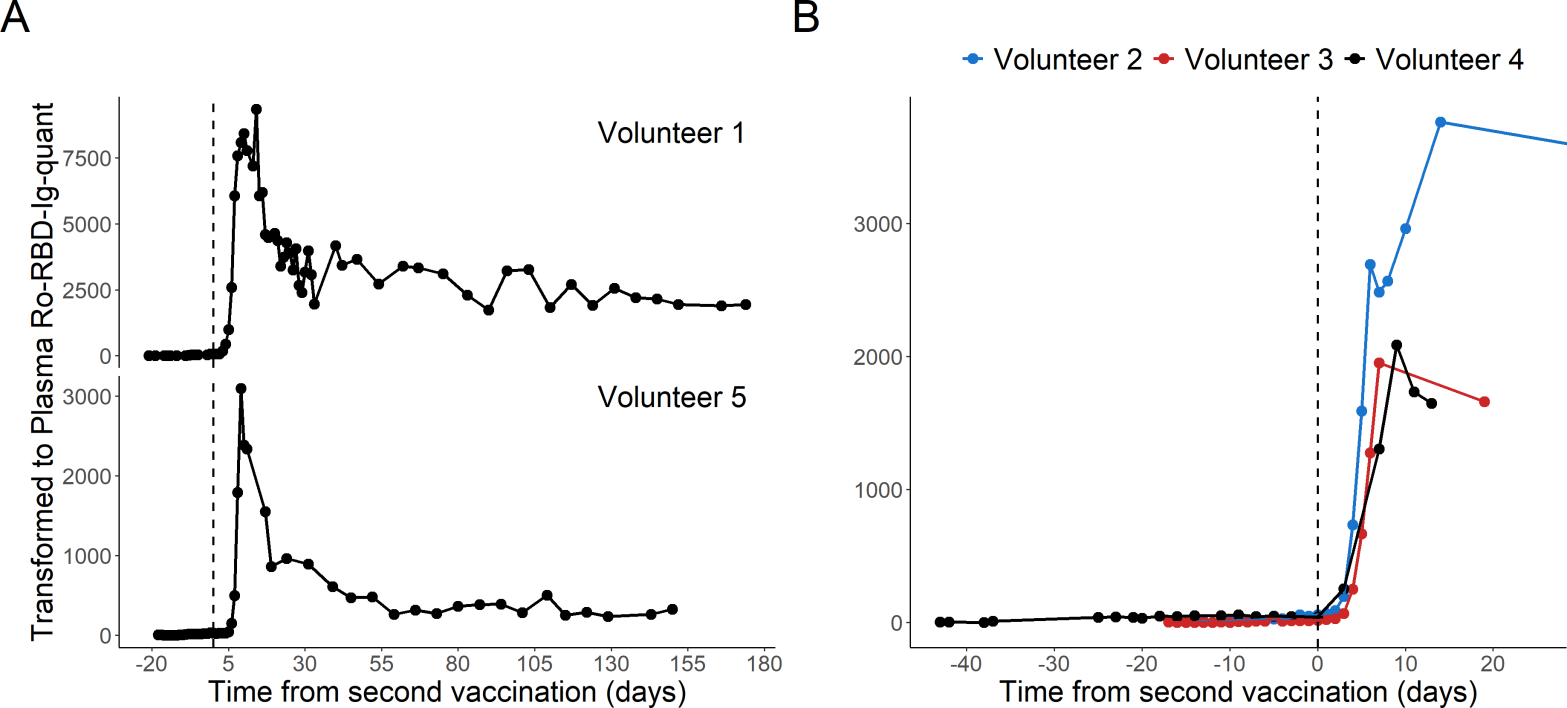
Temporal evolution of the anti-S titers in BNT162b2 vaccinated SARS-CoV-2 naïve subjects. The antibody response is shown in back-calculated venous sample BAU/mL. Vertical dashed lines indicate the date of the second vaccination, set to day 0. (**A**) Time series of two subjects over 150 days (Volunteers 1 and 5). (**B**) Titer-increase after the second dose in three other subjects (Volunteers 2, 3, and 4).

Additionally, we analyzed a set of DBS from subjects undergoing vaccination. In this group, five subjects self-sampled consecutive DBS during their vaccination with BNT162b2 and thereafter at random but frequent timepoints (sometimes daily). The DBS cards were anonymized and analyzed in retrospect. Time series are depicted in Fig. 4. It can be easily appreciated that the quantitative measurement from DBS eluates with the protocol described in this manuscript is able to track the temporal evolution of the titers with the rapid rise after the second vaccination, peak 1 week thereafter and then a biphasic drop over time.

**Fig 5:**
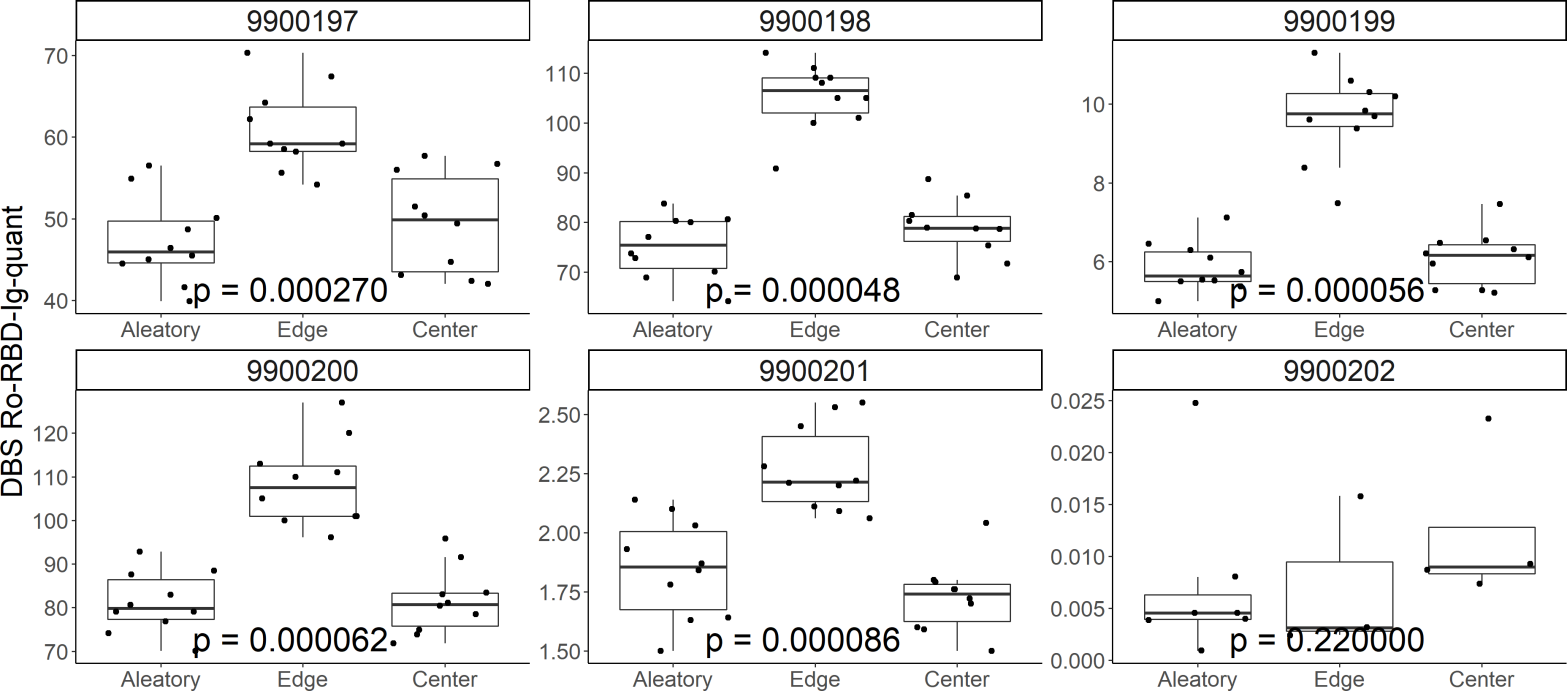
Distribution of DBS eluate values for six different repetitively punched DBS samples. Blood spots were punched on the drying edge (*coffee stain* middle boxplot) or the center (*volcano effect* right boxplot) of each respective blood spot. The same samples were also punched using the automatic system of the Panthera machines, aleatory (*aleatory*, left boxplot). For all the samples, except 99000202, significant differences could be detected by applying the Kruskal-Wallis test (*P* < 0·001), demonstrating higher values at the edges of the blood drop, which would be consistent with a slight coffee stain effect.

To investigate potential bias in the DBS punching as an explanation for variation, we performed the analysis of multiple DBS of different subjects grouped in edge, center and arbitrary as chosen by the automated puncher system (Fig. 5). Interestingly, the measurements in four out of five cases were significantly higher at the edge than at the center or the aleatory measurement (mean 27.7%/19.0%, median 31.7%/6.6%, min = −31.4%/−6.2%, max = 73.2%/96.7% of the aleatory signal, for center/edge, respectively). In the anti-N serology protocol, this phenomenon was found to be not significant (18). This effect has been described for different analyses and is called the coffee stain effect (23). Of note, the arbitrary punching as performed by the semi-automated puncher results in reliable values with little variation and not influenced by potential chromatographic effects; thus, this approach was chosen for the study (Fig. 5).

**Fig 6:**
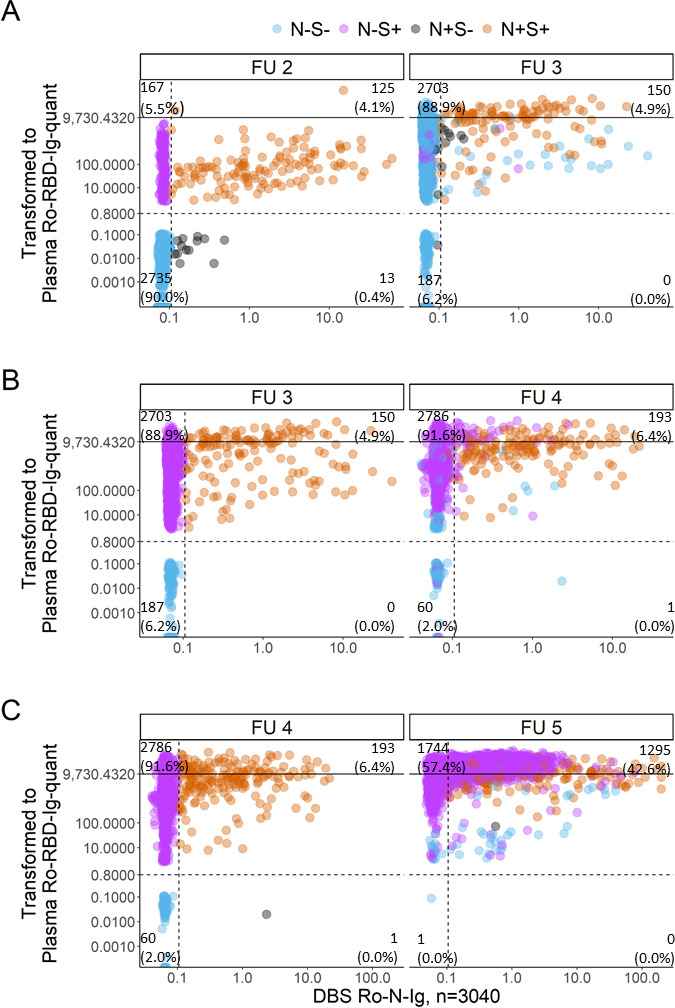
Scatterplot of four follow-ups of the KoCo19 cohort for people who participated in all rounds (*n* = 3,040). The Ro-N-Ig measurement from DBS is abbreviated with “N,” and Ro-RBD-Ig-quant from the same DBS is abbreviated “S.” Positivity is represented with “+,” negative, below cutoff with “−.” The color code is defined by the status of the respective subject in the (**A**) second, (**B**) third, and (**C**) fourth follow-up, respectively (represented by “FU”). Blue dots represent N−S−, orange dots represent *N* + S + , gray spots are N−S + and pink dots are *N* + S−, considering the left column as reference for color-coding. Samples above the nonlinear range of Ro-RBD-Ig-quant (solid black line at 9730.4 for back calculated plasma BAU/mL) were not diluted. (**A**) Evolution from second to third follow-up. Left: Second follow-up sampled between March and April 2021; right: Third follow-up sampled between July and September 2021. (**B**) Evolution from third to fourth follow-up, sampled between October and December 2021 and (**C**) evolution from the fourth to the fifth follow-up, sampled between May and July 2022.

Due to the similarity of the protocols, the parallel determination of SARS-CoV-2 anti-N antibodies and anti-S antibodies is possible from the same DBS eluate. This approach was used in the analysis of the representative population cohort in Munich, KoCo19 (19, 20). Results are shown exemplarily in Fig. 6 including only the participants that gave a sample in all the follow-ups (see Fig. S4 for raw value distributions for all participants). Here, anti-N and anti-S values are depicted in the x- and y-axis, respectively. It can be observed that with the start of the vaccinations in early 2021 (Fig. 6A follow-up 2), about 5.5% of the participants were positive in anti-S and negative in anti-N, which is in accordance with a vaccination titer and roughly also represents the fraction of persons eligible for full vaccinations at that time. Almost 4.1% of samples analyzed show a pattern expected of infected individuals with both values positive, while 0.4% were only positive in anti-N representing a group being either an anti-S non or late-responder after infection, or a false positive value for anti-N. Of note, most of these raw values are very low as compared to the anti-N values seen in the double positive group, considered as infected individuals. Thus, it is likely to be individuals shortly after acute infection which are not yet positive in anti-S but already show a beginning low anti-N reactivity. This is also supported by the fact that all but one of those individuals were found to be anti-N and anti-S positive in the subsequent later sampling round. Around 90% of the participants were still naïve to both SARS-CoV-2 vaccination and infection. Compared to the results of follow-up 2, in follow-up 3, a massive vertical upward shift of the data points can be observed. Between July and September 2021, the vaccination campaign was already under way for quite a long time among the Munich population. Therefore, 88.9% of the samples received in this study round show a result pattern expected in vaccinated individuals (anti-S positive and anti-N negative). Due to low SARS-CoV-2 spread during the summer and very little losses due to titer drop after infections, the rate of double positives increased slightly to 4.9%, with the increased anti-S values being caused by the combination of vaccination and infection. In parallel, the anti-N positive but anti-S negative population vanished as mentioned above, migrating to the double positive fraction, supporting that those subjects actually were only at the beginning of their seroconversion and developed their anti-S titer after follow-up 2. The amount of participants still naïve to both SARS-CoV-2 vaccination and infection decreased to 6.2%. After the summer of 2021, follow-up 4 (Fig. 6B) took place with the fraction of vaccinated people increasing even further to 91·6%. The amount of infected subjects slightly increased to 6·4% with 50/193 (25·9%) being newly infected. Of these 50 participants, 5/50 (10.0%) confirmed breakthrough infections since they became infected after vaccination (from S + N in follow-up three to S + N + in follow-up four, denoted in black). Only one participant was anti-N positive but still not seroconverted in anti-S. With the fifth follow-up (Fig. 6C), all participants despite one completed seroconversion. With the advent of Omicron, the proportion of anti-N positives increased dramatically to 42.6% (1,295/3,040), with 86.3% (1,118/1,295) of newly positives [96.2% (1,075/1,118) breakthrough infections, black dots]. The one participant positive in anti-N but negative in anti-S in follow-up four converted also in anti-N, suggesting also in this case a very early infection with the development of the anti-S titer after the follow-up.

## DISCUSSION

Recent published studies have highlighted the convenience of using DBS for tracking the seroprevalence for SARS-CoV-2 Spike protein in subjects (16, 17, 27). Therefore, the main objective of this study was to investigate the possibility of combining the qualitative anti-N DBS Roche Elecsys protocol published by our laboratory (18) with a quantitative anti-S measurement. To facilitate automated workup, we decided to use the Roche Elecsys anti-SARS-CoV-2 S assay for quantitative measurements (22, 28, 29). We already established the compatibility of the chemistry used in this study with the Elecsys system (18), in addition to a well-working elution protocol. Raw data showed a good separation between the positive and negative populations. Furthermore, the correlation between the values measured in DBS eluates and plasma was very high (Spearman’s rho = 0.95), and we could derive a formula to back-calculate anti-S titers from the values measured from DBS eluates with high confidence [$venousUnits=10^{1\cdot 623+0\cdot 986\cdot log_{10}DBSUnits}$, with BCA intervals of intercept and slope (1.590; 1.695) and (0.900; 1.022), respectively].

Quantitative measurements from DBS are difficult, and most of the studies fail to take into account the many different aspects that have to be considered (30). A certain limitation is the hematocrit effect or chromatographic artifacts such as the coffee stain or volcano effect (23). While we cannot control for the hematocrit effect easily, we investigated chromatographic effects. Hereby, unlike what we observed for the anti-N serology, a significant increase in titer values was seen especially at the edges compared to an aleatory puncher placement (in mean 27.7%/19.0% increase of the value compared to the aleatory measurement for center/edges, respectively). However, we are not using manual punching, but a semi-automated process with a punching robot placing the punching points at arbitrary positions within the DBS. The arbitrary method proved very stable over multiple punches. Thus, we conclude that the chromatographic effect, while present, is not influencing our results significantly, but only increasing variability slightly. One sample shown in Fig. 5 did not show the same pattern of distribution with increased reactivity on the edges of the DBS as observed with the other samples (Fig. 5; 9900202). This is most likely due to coagulation effects changing the chromatographic distribution of the blood in the filter paper card during application and drying. Either hyper- or hypocoagulability might affect the sample leading to a more even distribution of the antibodies on the DBS card. Due to arbitrary placement of the punches, however, no significant change in values results in such samples compared to those with chromatographic coffee stain effect, as can also be appreciated in the correlation between venous blood and DBS values (Fig. 2).

Overall, variability is low for a serological technique with a mean value signal difference of 1 (95% CI 0.26, 3.87; see Fig. 3). Also, only a few outliers were observed. Looking at the longitudinal data obtained from subjects undergoing vaccination, it can be easily appreciated that the de-facto variations introduced by all possible factors combined are comparably small and still allow to monitor the evolution of the titer in sufficiently high resolution for any clinical needs. The data obtained here are also in line with what was observed in other studies after mRNA-based vaccines (6). Actually, slight variations are also not critical for the clinical interpretation of titer values. The purpose of such assays is an estimation of the titer levels to assess vaccination success or previous infection with SARS-CoV-2. The dilution performed eluting the DBS discs results in lower overall raw values obtained in the measurement. This obviously brings values closer to the detection limit. While the manufacturer guarantees linearity between 0.4 and 250 U/mL (31), we observed that even at around 0.1, linearity is sufficient to allow for quantification, and the positivity threshold could be put at 0.115 still maintaining 96.63% sensitivity and 97.81% specificity. The data shown in this manuscript correlate with the values obtained between the venous plasma and the DBS eluates obtained from the same person on the same day. Obviously, this does not take into account any possible changes in antibody specificity away from the wild type version of nucleocapsid or spike/RBD as used in the assays of the manufacturer. This means that the DBS sample elution can only be as good as the assay used with it, so over time, the antigens used may be changed to reflect changing specificities of the serology in the population. So far, we did not observe any implausible results or tendencies pointing toward this being an issue. Nevertheless, further developments with updated omicron sequence-based antigens are currently performed.

### Summary

We present a protocol suitable for high throughput analysis for quantitative SARS-CoV-2 Spike serology. The protocol can be combined with the previously published anti-N serology. Due to the use of DBS as sample material, this protocol lends itself for use in large epidemiological studies and is in routine use in our laboratory since spring 2021. Samples can be collected at home without the need for any medical training in study participants. Also, the cards can be sent via regular mail service and can be stored for prolonged periods of time. Although slightly increased variation compared to serum/plasma samples has to be expected, the relevant quantitative information such as titer/Units can be reliably extracted making it a viable tool.

## Data Availability

Anonymized raw data or aggregated data as applicable will be made available upon qualified request to other research groups.

## SUPPLEMENTAL MATERIAL

Supplemental material spectrum.02885-23-s0001.pdf All supplemental figures with their respective captions.

ASM does not own the copyrights to Supplemental Material that may be linked to, or accessed through, an article. The authors have granted ASM a non-exclusive, world-wide license to publish the Supplemental Material files. Please contact the corresponding author directly for reuse.
