# Supplementary material for: A Dried Blood Spot protocol for high-throughput quantitative analysis of SARS-CoV-2 RBD serology based on the Roche Elecsys system
Source: Microbiol Spectr. 2024 Mar 1;12(4):e02885-23. doi: 10.1128/spectrum.02885-23 (PMC10986497; doi:10.1128/spectrum.02885-23)
Supplement: Supplemental material — All supplemental figures with their respective captions. [file spectrum.02885-23-s0001.pdf]

SUPPLEMENTAL MATERIAL TO:

A Dried Blood Spot Protocol for high-throughput quantitative analysis of SARS-CoV-2 RBD Serology based on the Roche Elecsys System

Noemi Castelletti,*[a,b,e] Ivana Paunovic,*[a,e,f] Raquel Rubio-Acero,*[a] Jessica Beyerl,[a,e,f] Michael Plank,[a] Christina Reinkemeyer,[a] Inge Kroidl,[a] Ivan Noreña,[a] Simon Winter,[a] Laura Olbrich,[a] Christian Janke,[a] Michael Hoelscher,[a,c,d,e] Andreas Wieser,[a,c,e,f]# on behalf of the KoCo19/ORCHESTRA Working group

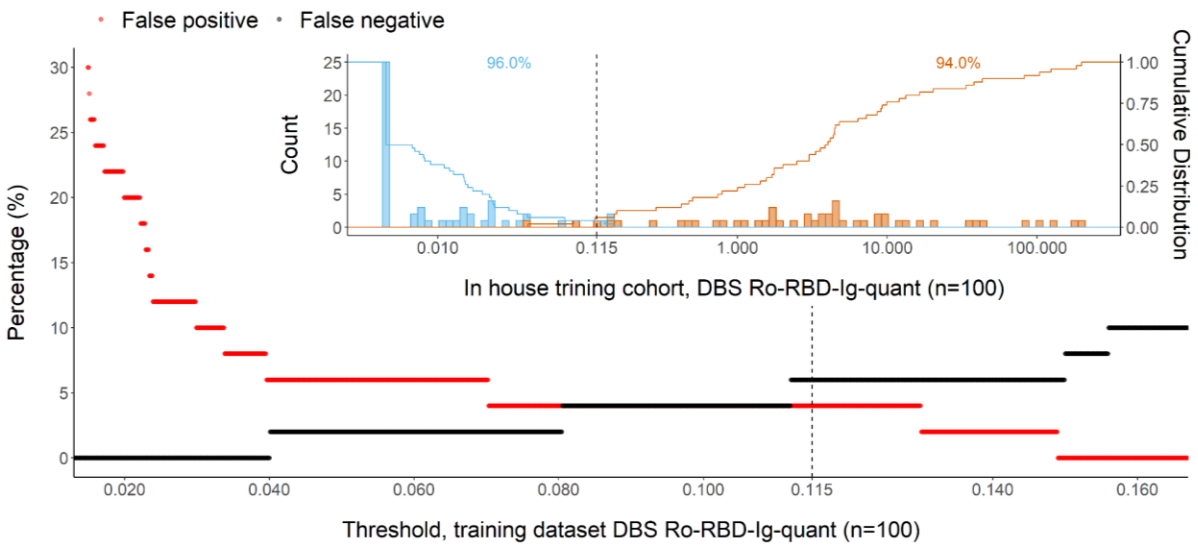

Supplemental Figure 1: Establishment dataset (n = 100, 50 positives) for cutoff estimation plotted as percentages of false positives/negatives depending on a variable threshold for DBS. The insert above right shows the frequency distribution of the detected antibody units detected in SARS-CoV-2 vaccinated or naturally infected individuals in DBS eluates. The dashed vertical line denotes the empirically determined cutoff value (0·115) for result classification.

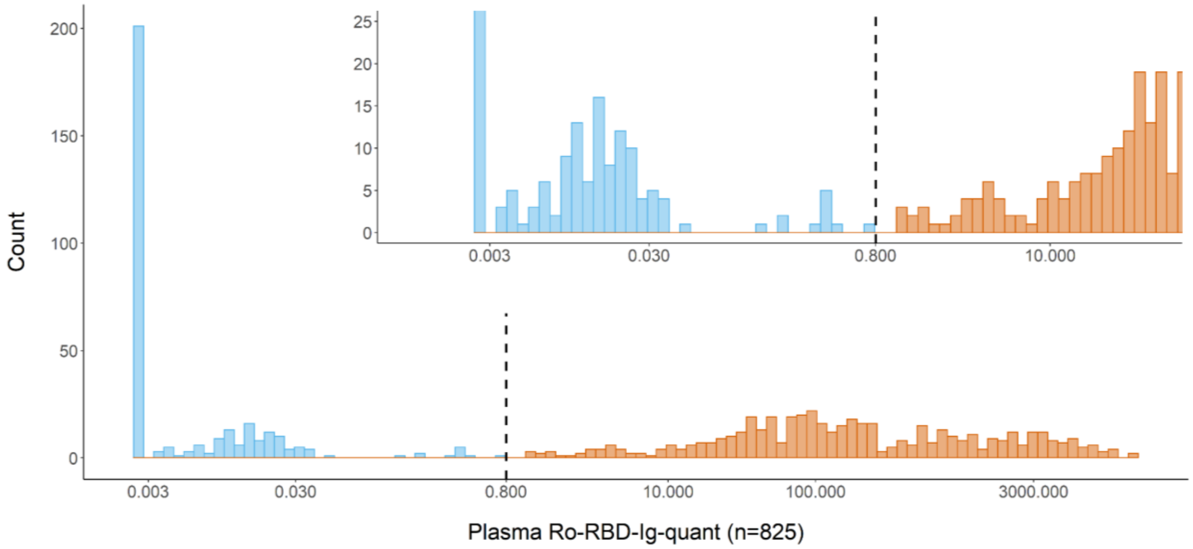

Supplemental Figure 2: Frequency distribution of antibody units detected in SARS-CoV-2 vaccinated or naturally infected individuals. Plasma eluates from patients of our in-house KoCo19 cohort and volunteer donors were used (n = 825). The dashed vertical line denotes the manufacturer's cutoff value (0·8) for result classification. The insert in the top right represents a zoom-in on the y-axis to allow better visualisation of the cutoff region.

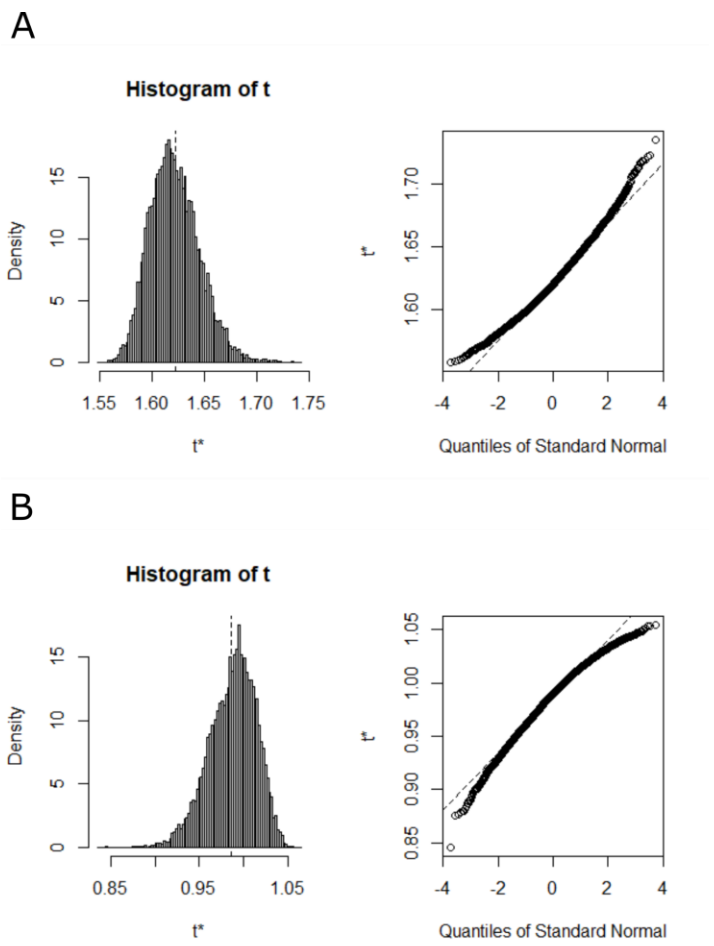

Supplemental Figure 3: Examination of the model results for bootstrapped linear regression of plasma values on DBS eluate, both variables log10 converted. The bootstrapped estimate is based on 10000 replications. Density distribution (left) and quantiles of the standard normal distribution (right) are presented for both significant parameters intercept **(A)** and slope **(B)**.

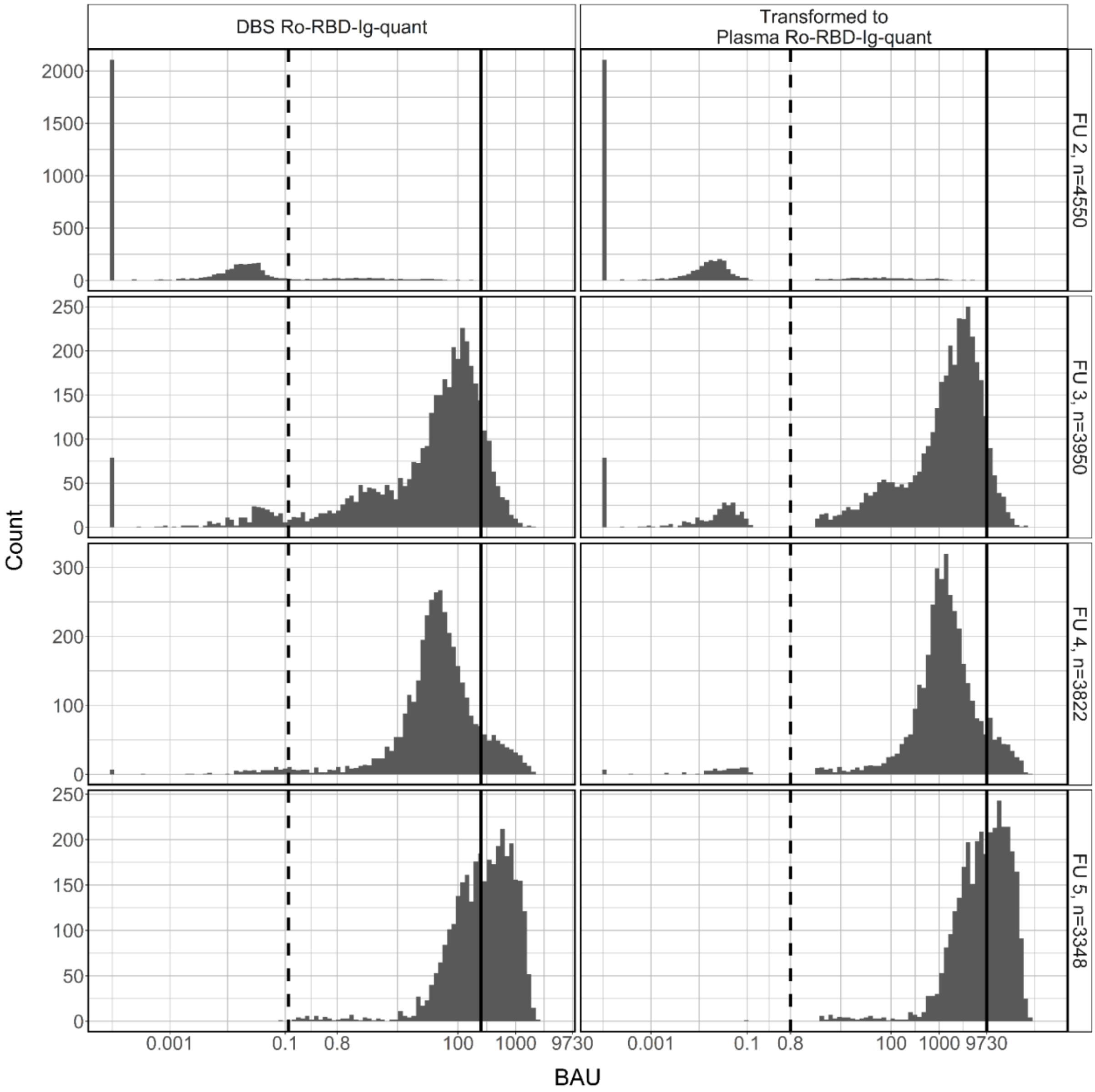

Supplemental Figure 4: Frequency distribution of detected antibody titre generated by SARS-CoV-2 vaccines and infections from patients of our in-house KoCo-19 cohort in the follow-ups (FU, rows of the plot) two to five. Samples above the nonlinear range (250 for DBS and 9730·4 for transformed to plasma) were not diluted. (**Left column**) DBS eluates values, (**Right column**) to plasma transformed values. The dashed vertical line denotes the (**Left column**) empirically / (**Right column**) manufacturer's determined cutoff value for result classification.

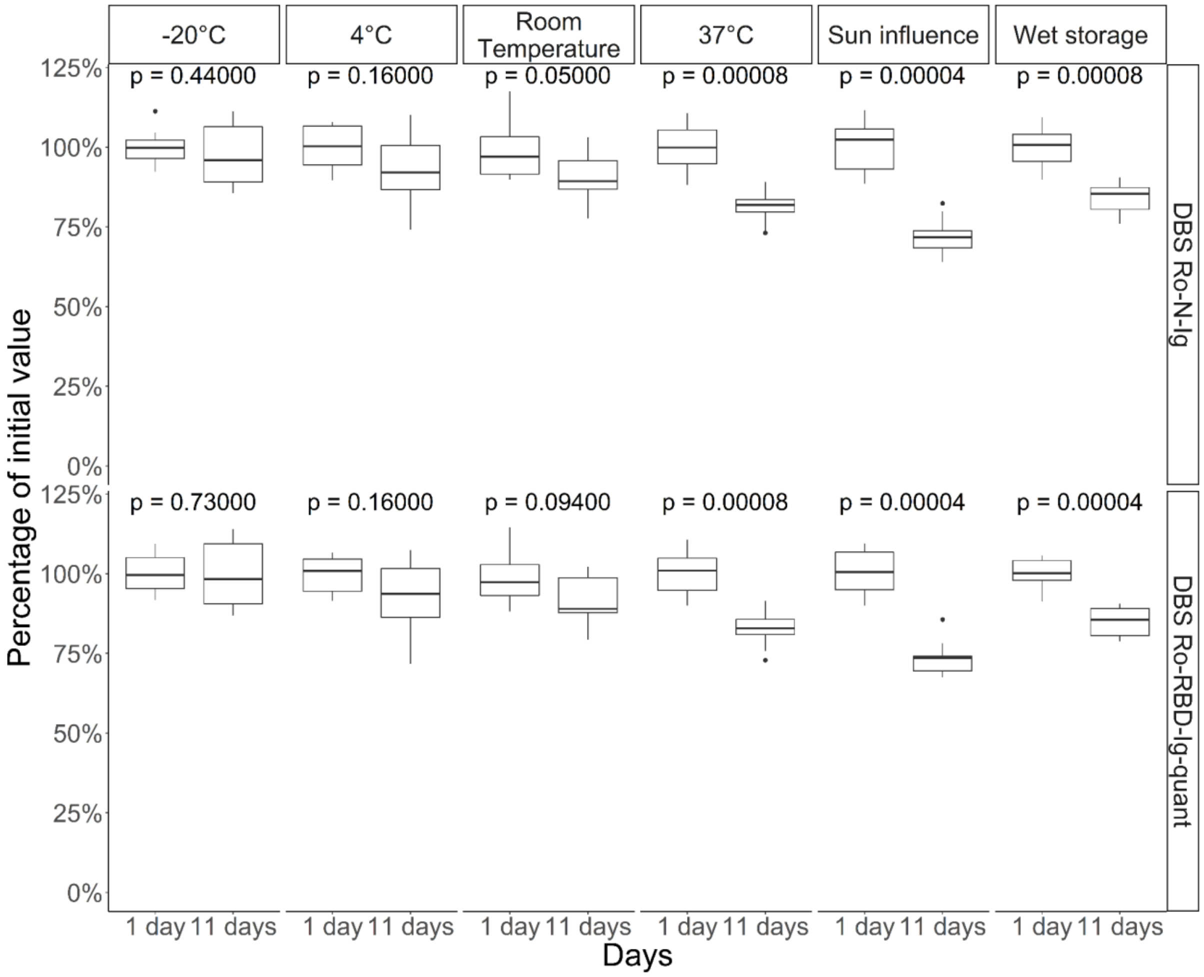

Supplemental Figure 5: Decay characteristics of DBS from three different subjects performed in individual triplicates over time. Punching and analysis was performed the same day at the indicated intervals. Wet storage: DBS were dried for 3h only and packed wet into a zipper bag. They were stored wet at room temperature and only taken out of the zipper bag for punching and re-inserted immediately. Here punching was producing errors due to the paper being floppy and wet. Sun influence: DBS was dried for 12h and then packed. The DBS was left on the window board in direct sun for the following day. Rest of the storage time was at room temperature (RT) conditions. Fridge condition (4-7°C): samples were dried at room temperature and then packed in zipper bags. The sample was stored in the fridge. Hot condition (36-38°C): samples were dried at room temperature and then packed in zipper bags. Zipper bags were then stored in the incubator and only taken out for punching. Before re-inserting into the incubator, DBS were re-packed to avoid condensation.
